# The Neural Code for Taste in the Nucleus of the Solitary Tract of Rats with Obesity Following Roux-En-Y Gastric Bypass Surgery

**DOI:** 10.3390/nu14194129

**Published:** 2022-10-04

**Authors:** Olga D. Escanilla, Andras Hajnal, Krzysztof Czaja, Patricia M. Di Lorenzo

**Affiliations:** 1 Department of Psychology, Binghamton University, P.O. Box 6000, Binghamton, NY 13902, USA; 2 Department of Neurobiology and Behavior, Cornell University, Ithaca, NY 14853, USA; 3 Department of Neural and Behavioral Sciences, College of Medicine, The Pennsylvania State University, Hershey, PA 17033, USA; 4 Department of Biomedical Sciences, College of Veterinary Medicine, University of Georgia, Athens, GA 30602, USA

**Keywords:** Roux-en-Y gastric bypass, taste, nucleus of the solitary tract, neural coding

## Abstract

Previous work has shown that taste responses in the nucleus tractus solitarius (NTS; the first central relay for gustation) are blunted in rats with diet-induced obesity (DIO). Here, we studied whether these effects could be reversed by Roux-en-Y gastric bypass (RYGB) surgery, an effective treatment for obesity. Rats were fed a high energy diet (60% kcal fat; HED) both before and after undergoing RYGB. Electrophysiological responses from NTS cells in unrestrained rats were recorded as they licked tastants from a lick spout. Sweet, salty, and umami tastes, as well as their naturalistic counterparts, were presented. Results were compared with those of lean rats from a previous study. As with DIO rats, NTS cells in RYGB rats were more narrowly tuned, showed weaker responses, and less lick coherence than those in lean rats. Both DIO and RYGB rats licked at a slower rate than lean rats and paused more often during a lick bout. However, unlike DIO rats, the proportion of taste cells in RYGB rats was similar to that in lean rats. Our data show that, despite being maintained on a HED after surgery, RYGB can induce a partial recovery of the deficits seen in the NTS of DIO rats.

## 1. Introduction

Approximately 70% of US adults are classified as overweight, with ~37% reaching clinical obesity [1]. Treatments that include modifications to diet and sedentary lifestyle are often ineffective, leading to weight-cycling [2,3,4]. Currently, the most effective weight-loss method in terms of both magnitude and permanence is bariatric surgery, specifically, Roux-en-Y gastric bypass (RYGB [5]).

The primary goal of RYGB surgery is to promote a negative energy balance via restriction of energy intake. This is accomplished by two ways. First, the stomach is configured into a smaller gastric pouch, decreasing the volume of food the stomach is able to hold. Secondly, nutrient absorption is reduced by connecting the reduced stomach distally to the jejunum, bypassing the duodenum and proximal jejunum. However, the reduced stomach size and re-routing of the small intestine have effects beyond gastric motility and nutrient absorption. For example, the reconfiguration of the stomach transects vagal afferents [6] resulting in synaptic reorganization of central gustatory structures such as the nucleus of the solitary tract (NTS [7]). Furthermore, the earlier discharge of undigested food and intraluminal content, due to the shortened upper intestinal path, changes gut–brain hormonal signaling. 

The stomach and small intestine are organs with intricate control of hormonal systems influencing neuronal circuits governing ingestive behaviors. After RYGB surgery, hormones released from the gastrointestinal tract that promote satiety (e.g., glucagon-like peptide-1 (GLP-1) and peptide YY), remain elevated [8,9], whereas hormones that promote hunger (e.g., ghrelin) are decreased [10]. While the taste system is the final arbiter of consumption, gut-related hormones have a reciprocal relationship with the neural coding and perception of taste. For example, hormones that enhance post-prandial satiety generally decrease sweet-, umami-, and fat-taste responsiveness [11,12], whereas hormones that promote hunger enhance taste responsiveness (for review on taste perception and hormonal modulation; see [13,14]. Additionally, RYGB reverses the loss of responsiveness to satiety-promoting hormones in neurons in the caudal brainstem, further exaggerating this effect [15]. 

Post-RYGB patients often undergo significant changes in taste perception. For example, patients often show a short-term increase in sweet-taste detection and an overall decrease in sweet-taste preference [16], decreased preference for fatty foods [17], and shifted preferences from processed foods towards fruits and vegetables [18,19]. These changes in taste preferences ultimately beg the question of how neurons process taste as information comes into the central nervous system after RYGB. 

The NTS, the first synapse in the central gustatory system, is an attractive target for the study of taste-processing changes in animals with RYGB. In addition to receiving oral-sensory (taste, temperature, texture, etc.) input in the rostral pole, the NTS receives vagal afferents via innervation of the gastrointestinal tract in its caudal pole, with a high degree of intranuclear connectivity [20,21,22]. Neurons located within the NTS express a wide array of receptors involved in metabolism (e.g., CCK, GLP-1, etc. [23,24]). In effect, the NTS is unique in that it is a structure with multiple levels of information regarding internal metabolic state and the bioavailability of macronutrients from foodstuffs. 

Here, we studied how taste-evoked responses in single neurons within the NTS change in awake, freely licking rats with obesity following RYGB surgery. Rats were maintained on a high-energy diet (HED) both before and after RYGB surgery to separate the effects of the surgery from the effects that might have accompanied RYGB-induced weight loss. We hypothesized that RYGB surgery would reverse the alterations in NTS taste responses that have been reported in rats with diet-induced obesity (DIO). Specifically, DIO rats had NTS taste responses that were smaller in magnitude, shorter in duration, and longer in latency than those in lean rats [25]. Results confirmed that NTS taste responses after RYGB surgery partially recovered the changes observed in DIO rats; lingering deficits in NTS taste responses might be attributed to the effects of an HED. 

## 2. Materials and Methods

### 2.1. Subjects

Male Sprague Dawley rats (*n* = 35) served as subjects. Of 40 rats that were shipped to Binghamton University following RYGB surgery, there were three rats that did not survive electrode implantation surgery. Of the remaining 37 rats, 17 provided isolated NTS cells that could be included in the analyses. Animals undergoing RYGB surgery were maintained on a high-energy diet (HED; 60% kCal from fat, 20% kCal carbohydrates, 20% kCal protein; Research Diets D12492, New Brunswick, NJ, USA) for at least eight weeks prior to RYGB surgery and for the remainder of the experiment. Following recovery from RYGB surgery, rats were shipped to Binghamton University, where they were quarantined for two weeks prior to electrode implantation. All rats were pair-housed until microelectrode implantation, after which they were singly housed. Housing was in a temperature-controlled vivarium with a reverse 12-h light–dark schedule (lights on at 21:00). Food was available ad libitum and water was available at least one h/day. Animals were weighed every week to monitor general health. 

RYGB surgical procedures were approved by the Institutional Animal Care and Use Committee (IACUC) at The Pennsylvania State University, College of Medicine, Hershey, PA, USA. The surgeries were conducted under the protocol entitled “Vagal influence on vagal plasticity and neural coding of taste” (IACUC Protocol #: PRAMS201647305, 2015–2021). Additional experimental procedures, including electrode implantation and electrophysiological recording, were approved by the IACUC at Binghamton University (Protocol title: Temporal coding in the gustatory system of the rat; Protocol #: 735-15, 2015–2018). All procedures were in accord with the National Institutes of Health Animal Welfare Guide.

Lean male rats (*n* = 12) that were part of previous experiment [25] served as controls. 

### 2.2. RYGB Surgery 

All 35 rats underwent RYGB surgery. The techniques and perioperative care were previously described [26]. Rats were fasted overnight and had water ad libitum prior to surgery, then anesthetized (isoflurane: 3% for inductions, 1.5% for maintenance). All animals were pretreated with antibiotic (gentamycin: 2.5 mg/kg, IM, APP Pharmaceuticals, LLC, Schaumburg, IL, USA and ceftriaxone: 30 mg/kg, SC, Sandoz Inc., Princeton, NJ, USA) and Buprenex (buprenorphine, 0.05 mg/kg, SC, Reckitt Benckiser Pharmaceuticals, Richmond, VA, USA) for pain control. Utilizing a sterile procedure during the surgery, through a midline laparotomy, the stomach was separated in the RYGB procedure using a linear-cutting stapler (ETS-Flex Ethicon Endo surgery, 45 mm blue load) to create a small gastric pouch isolated from the bypassed stomach. The jejunum was measured 15 cm from the ligament of Treitz, and the distal segment was anastomosed end-to-side to form a pouch gastrojejunostomy. The proximal jejunum was anastomosed 15 cm along the distal limb end-to-side. Both anastomoses were created utilizing interrupted 5-0 monofilament suture material (Prolene) sutures. The muscle layer was closed using running 3-0 nylon suture, and the skin layer was closed utilizing running 4-0 nylon suture. Postoperative care consisted of normal saline (10 mL bid, SC), ceftriaxone (30 mg/kg, SC), gentamycin (2.5 mg/kg, IM, USA), and carprofen (5 mg/kg, SC) for 3 days. Animals received BOOST (Nestle Nutrition, Minneapolis, MN, USA) 24 h after surgery for 5 days with water ad libitum, then returned to their designated HED. Efficiency and homogeneity of individual rat’s responses to the surgery was confirmed based on body weight outcomes screened daily for the first two postoperative weeks and weekly thereafter, as well as changes in body composition (see next section). 

### 2.3. Body Composition Determination

At the end of the experiment, all rats underwent a Dual-energy X-ray Absorptiometry (DXA) scan to determine body composition. To perform DXA scans, animals were lightly anesthetized with Dormitol (medetomidine HCl, 0.1 mg/kg, SC, Pfizer, Inc., New York, NY, USA). They were then placed on a scanning bed where body composition was calculated by Hologic APEX Discovery A software (Hologic, Bedford, MA, USA). Scan results allowed the determination of both body fat and lean tissue mass. After DXA scanning was complete, anesthesia was reversed by Antisedan (atipamezole, 0.1 mg/kg, IP, Pfizer, Inc., New York, NY, USA), and animals were returned to their home cage. 

### 2.4. Microelectrode Construction

Details of how the microwire assemblies were constructed are described elsewhere [27]. Briefly, a bundle of eight 25 µm tungsten wires insulated with Formvar (California Fine Wire, Grover Beach, CA, USA) were soldered to an Omnetics connector (CON/8o50m-10P; Plexon, Inc., Dallas, TX, USA) and threaded through a polyimide tube (FHC, Inc., Bowdoin, ME, USA). The tips of the microwires were staggered over approximately 1 mm. Just before implantation, the exposed microwire bundle was dipped in a sucrose–gelatin mixture and allowed to dry. A stainless-steel wire (320 µm) was wrapped around a skull screw during surgery and served as ground.

### 2.5. Microelectrode Implantation Surgery

All rats were given an injection of Buprenex (buprenorphine, 0.05 mg/kg, SC) about 1 h prior to surgery. Anesthesia was induced with 3% isoflurane in oxygen. A surgical level of anesthesia was maintained with 1–2% isoflurane in oxygen. Vital signs (blood oxygenation, blood perfusion, heart rate, and respiration rate) were monitored using a PhysioSuite MouseSTAT sensor (Kent Scientific, Torrington, CT, USA). Body temperature was maintained at 37 °C using a rectal thermometer connected to an auto-regulating heating pad (Model No. 40-90-8D, FHC, Bowdoin, ME, USA). Artificial tear gel (AltaLube, Altaire Pharmaceuticals, Aquebogue, NY, USA) was applied to the eyes to prevent drying. The crown of the head was shaved, and the animal was then placed in the stereotaxic apparatus (Model 1900, Kopf, Tujunga, CA, USA) with the head angled 25° downward. The head was swabbed three times with betadine and 95% ethyl alcohol, and an incision was made in the scalp along the midline. The fascia and muscle were then retracted by blunt dissection. Six skull screws were secured to the skull as anchors, with one serving as ground. A hole was drilled above the NTS (15–16 mm caudal, 1.5–2.5 mm lateral to bregma), and the dura was resected. The microwire assembly was lowered slowly into the brain until the tip was at 5.5–6.5 mm below the brain surface. As the electrodes were being lowered, the tongue was bathed periodically with NaCl (0.1 M) followed by artificial saliva (AS) rinse while the electrophysiological activity was monitored for the presence of a taste response. The microwire assembly and skull screws were then embedded in dental acrylic. Once the acrylic hardened, the rat was given 100% oxygen until its heart rate reached ~400 bpm. It was then removed from the stereotaxic instrument and placed on a warmed surface until it recovered from anesthesia and was fully mobile. 

Postoperatively, buprenorphine HCl (0.02 mg/kg, s.c.) and gentamicin (6 mg/kg, s.c.) were given as an analgesic and antibiotic, respectively, for three days. Topical antibiotic (Neosporin) was applied around the head-cap daily for five days to help prevent infection. Animals in post-operative care were also given sterile isotonic saline or a lactated ringer’s solution (5–10 mL s.c.) to help replenish any lost fluids and prevent dehydration if needed. Animals were kept in post-operative recovery until they began to gain weight. 

### 2.6. Taste Stimuli

Taste stimuli included both prototypical taste stimuli and naturalistic taste stimuli [28]. Naturalistic stimuli were included because it has been shown that responses to these more complex tastants convey more information about taste quality than traditional pure chemicals [28]. Prototypical taste stimuli included: 0.5 M sucrose (High Sucrose, HS), 0.1 M sucrose (Medium Sucrose, MS), 0.05 M sucrose (Low Sucrose. LS), 0.1 M NaCl (High NaCl, HN), 0.05 M NaCl (Low NaCl, LN), 0.1 M monosodium glutamate + 0.01 M inosine monophosphate (MSG). These solutions were made from reagent grade chemicals purchased from Sigma-Aldrich (St. Louis, MO, USA) or Fisher Scientific (Pittsburg, PA, USA). Unpalatable taste stimuli, including bitter and sour tastants, were excluded since pilot tests showed that when these animals encountered unpalatable tastes in the experimental chamber, they stopped licking for the remainder of the session. Naturalistic tastants included 100% grape juice (High Grape Juice, HGJ), 25% grape juice (Medium Grape Juice, MGJ), 12% grape juice (Low Grape Juice, LGJ), 75% clam juice (CJ; ~0.12 M NaCl, comparable to High NaCl), 25% cream (Cream, Cr). All stimuli were dissolved in artificial saliva (AS; 0.015 M NaCl, 0.022 M KCl, 0.003 M CaCl_2_; 0.0006 M MgCl_2_ [29,30]). AS was used as a “taste” stimulus and as a rinse.

### 2.7. Testing

Electrophysiological testing was conducted in an operant chamber (MED Associates, St. Albans, VT, USA), where rats were able to move freely. The stimulus delivery system consisted of twelve pressurized (~11 psi room air) 35 mL tastant reservoirs, each connected to a 20 ga stainless steel tube that delivered 12 μL of fluid when the rat broke the infrared beam by licking. Each tastant was routed through a computer-controlled solenoid (Parker-Hannifin, Fairfield, NY, USA). The stimulus delivery protocol was as follows: Each tastant trial consisted of five consecutive stimulus licks, followed by five AS rinse licks presented on a variable ratio 5 (VR5) schedule. That is, each rinse lick was separated by four to six dry (non-reinforced) licks. Taste stimuli were presented in a pseudorandomized order.

Prior to testing, animals were water-deprived for 22 h. Animals were placed in the experimental chamber and attached to a cable for electrophysiological recording. A house light inside the chamber signaled the beginning of a recording session, which lasted 30 min to one hour. After the recording sessions, animals were returned to their home cages and given one hour of water access.

Neural activity (25 µs resolution), as well as timestamps for licks, were recorded using Sort Client software (Plexon, Dallas, TX, USA). Waveforms were exported to Offline Sorter (Plexon, Inc., Dallas, TX, USA) for offline analyses. Single units were isolated based on the presence of distinct clusters in principal component feature space and a refractory period of >2 ms [31]. In some cases, more than one unit was recorded on the same day from different electrodes. To ensure that we did not consider duplicate units in our analyses, we applied a cross-correlation function (CCF) to all possible pairs of units on a given day. Those pairs of units that showed a narrow peak at exactly zero in the CCF were classified as the same unit and only one recording was included in subsequent analyses.

### 2.8. Data Analyses

Spontaneous firing rate was determined by obtaining the mean and standard deviation of firing rate (in spikes/s; sps) from multiple 10 s samples during which there was no licking. Baseline firing rate was calculated as the average firing rate (in sps) during the 1 s of activity before the first taste stimulus lick across all trials. Baseline firing rates reflected the firing rate when the rat was licking without reinforcement, i.e., dry licks.

Taste responses were observed to occur over two timescales: 5-Lick and Lick-by-Lick. Some cells showed responses to tastants that occurred following each lick, but significant responses were not apparent when activity was assessed over the 5-Lick period. Different analytical methods were used to detect each type of response.

5-Lick Taste Response: When analyzing taste responses after subjects received five consecutive reinforced licks (5-Lick), responses were determined with the use of a change point analysis that was applied to the peristimulus time histogram (PSTH) data for each tastant. Spike trains for each tastant delivery trial were aligned, with *t* = 0 s corresponding to the first tastant delivery, with a window extending from *t* = −2 to *t* = 4 s in 100 ms bins (total bins = 60). For each tastant, the mean spike rate was calculated ($\bar{x}$), then the cumulative difference from the mean for each PSTH bin was calculated ($x_{i}-\bar{x}$). The cumulative difference from the mean was then obtained by starting at 0 and summing the value of each successive bin $\sum_{i=1}^{n}x_{i}-\bar{x}$, and the value of the summation recorded for each bin i (note: at i=n this summation will always be equal to 0). To determine if a significant change occurred, a bootstrap test with *n* = 1000 samples was performed. For each bootstrap sample, the PSTH bins were randomly shuffled (sampled without replacement 60 times), and the cumulative difference from the mean calculated. For the observed PSTH data, the magnitude for comparison in the bootstrap test was taken to be the maximum value of cumulative difference from the mean, minus the minimum (i.e., the value of n resulting in the maximum and minimum value of ($\sum_{i=1}^{n}x_{i}-\bar{x}$). This value was additionally calculated for each bootstrap sample. Significance was calculated by finding the percentage of bootstrap samples that had a greater difference magnitude value than the observed sample. Once it was determined that a significant change had occurred, the change time was estimated by minimizing the mean squared error (MSE). This was obtained by splitting the data into two sections and calculating the sum of the MSE before and after the split (MSE_1_ + MSE_2_, where MSE_1_ =$\frac{1}{s}\sum_{i=1}^{s}x_{i}-\bar{x}$, and MSE_2_=$\frac{1}{n-s}\sum_{i=s+1}^{n}(x_{i}-\bar{x})$. This was done for each possible value of *s*, and the minimum value *s*_min_ was taken to be the bin before the change, while the value for *s*_min_ + 1 was the first bin after the change. Once change significance and change time were established, the PSTH was split at the change point, and the analysis was repeated recursively on each of the resulting segments; this process was repeated until no additional significant change points were found. In the period of the PSTH from *t* = −2 to *t* = 0 (termed “baseline”), any change points found were removed. Responses were considered to start from the first changepoint and continue until either a second changepoint was found or the end of the PSTH window was reached. If a third changepoint was found, a second response was considered to occur from the third changepoint to either the end of the window or a fourth changepoint. For each response found, response magnitude was calculated by adjusting for baseline (i.e., taking the mean spike rate during a response and subtracting the baseline spike rate).

Lick-by-Lick Taste Response: To assess Lick-by-Lick responses, a PSTH was generated with each tastant lick aligned at *t* = 0. Additionally, a PSTH comprised of each dry lick preceding a 5-Lick tastant trial was created, with each dry lick aligned at *t* = 0. The analysis was performed from 0 to 150 ms, with a bin size of 15 ms, using a one-way chi-squared goodness-of-fit test. The dry lick PSTH served as the expected values, while the tastant PSTHs served as the observed counts. For each test, the dry lick PSTH was normalized to have a sum equal to the relevant tastant PSTH. Prior to performing the chi-square test, if less than 80% of the expected values were greater than 5, one such bin was merged with the following bin. This process continued until either 80% or more of expected values were greater than 5, or there were only two bins left. Throughout this process, the corresponding observed bins were also merged. Finally, the chi-squared test was performed using the stats.chisquare function from Python’s scipy library.

### 2.9. Analyses of Licking

For each cell, we measured the extent to which its firing pattern varied with the lick cycle, called “lick coherence.” This was calculated using the Coherence Analysis function in NeuroExplorer 5.201 (NexTechnologies, Colorado Springs, CO, USA). Single-taper Hann windowing was used to calculate the values of 256 frequency bins between 0 and 50 Hz frequency with a 50% overlap between windows. Confidence intervals were calculated as described in [32]. Neurons with a coherence value above the 99% confidence interval for frequencies between 4 and 9 Hz were considered lick-coherent. 

To determine whether the lick pattern for each tastant was altered by RYGB surgery, we measured the interlick intervals for each tastant trial, the number of pauses during each tastant trial, and the total time to complete all five licks of a taste stimulus trial.

### 2.10. Histology

To confirm the location of an electrode, animals were deeply anesthetized with a lethal drug provided by animal care staff (Fatal Plus, Vortech Pharmaceuticals, Dearbon, MI, USA). Direct current (5 mA; 5 s duration) was passed through the headcap pin that corresponded to the channel that a taste-responsive neuron was recorded from to create an electrolytic lesion. Animals were then perfused transcardially with isotonic saline followed by a 10% neutral buffered formalin solution. Brains were extracted and stored in 10% formalin. Before sectioning, brains were cryoprotected with 30% sucrose in phosphate buffer solution for at least 24 h, after which they were sectioned into 30 μm slices and stained with cresyl violet.

## 3. Results

### 3.1. Body Weight and Composition

On the day of surgery, the mean body weight of the rats was 599 ± 10.9 gms. Postoperative weight changes over the three- to four-week recovery period showed RYGB surgery resulted in an average weight loss of 18% (ranging from 24% to 13%). Most of this weight loss was maintained consistently across animals, despite the fact that the animals were fed an HED (percent body weight relative to pre-operative weight: postop. Week 1, 832 ± 2.3%; post-op week 2, 84.5 ± 1.5%; post-op week 3, 92.08 ± 1.3%). This weight loss is consistent with those previously reported by our lab for dietary obese rats, i.e., Sprague Dawley male rats fed an HE [33,34] or genetic obese rats fed a normal chow diet [15], as well as reports from other laboratories with slightly varying rodent models of RYGB (e.g., [35,36]).

**Figure 1:**
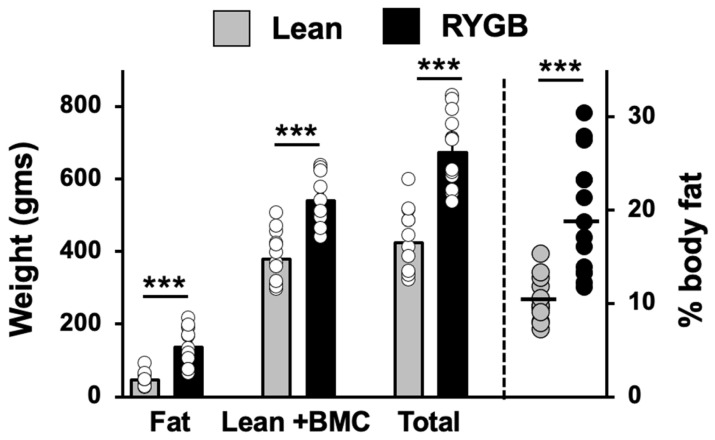
Body composition of Lean (*N* = 13) and RYGB (*N* = 14) rats measured at the end of the experiment. BMC = bone mineral mass. *** *p* < 0.001.

Analysis of body composition in RYGB rats and lean rats with taste responses showed that RYGB rats fed an HED had a significantly different body composition than lean rats fed a standard chow diet (Figure 1). Specifically, RYGB rats weighed more than lean rats (RYGB mean weight = 664.4 ± 29.2 gms; lean mean weight = 426.0 ± 26.9 gms; *t*(24) = 5.93, *p* < 0.001) and had more body fat (RYGB fat = 131.6 ± 15.1 gms; lean fat = 46.0 ± 5.8 gms; *t* (24) = 4.97, *p* < 0.001). Lean body mass plus bone mineral content (BMC) was also significantly greater in RYGB rats compared with lean rats (RYGB lean body mass + BMC = 533.1 ± 19.2 gms; lean body mass + BMC = 380 ± 21.6 gms; *t* (24) = 5.31, *p* < 0.001). Accordingly, RYGB rats had a significantly higher percent body fat than lean rats (RYGB percent body fat = 19.2 ± 1.7; lean percent body fat = 10.4 ± 0.7; *t* (24) = 4.49, *p* < 0.001). Comparisons were made using multiple unpaired *t* tests with a Holm–Sidak correction for multiple comparisons. Data were missing for two RYGB and one lean rat.

### 3.2. Responses to Taste Stimuli

Taste responses were recorded from 47 cells in 17 RYGB rats and 69 cells in 12 lean rats. An additional 344 cells in RYGB rats (total *N* = 391) and 367 cells in lean rats (total *N* = 436) that were not responsive to taste stimuli were also recorded. The mean number of taste-responsive cells recorded per animal in RYGB rats was 2.6 ± 0.4 (median = 2.5; range 1–6) and 5.8 ± 2.4 (median = 2.5; range 1–30) in lean animals. One lean rat had taste-responsive cells that were recorded from nine separate channels over 12 days. Data from lean rats were part of a previously published study [25]. They were re-analyzed with the same criteria for taste responsiveness as were applied to cells from RYGB rats to make direct comparison with RYGB results. Using these new criteria, response measures in lean rats in the current study correlated 0.95 with response measures used in [25]. However, there were fewer rats with taste-responsive cells in the present study since some cells in [25] only responded to tastants that were not tested here.

**Figure 2:**
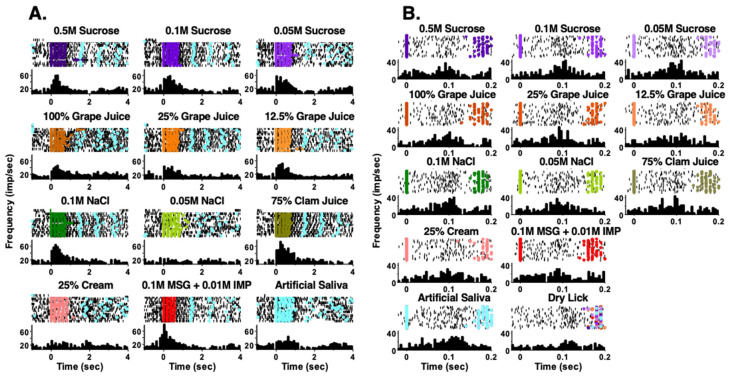
Examples of NTS responses in RYGB rats that occur in two time scales. In both (**A**,**B**), the top of each panel shows a raster of the firing of the cell. Each row represents one trial. Black dots indicate a spike and colored dots indicate reinforced licks. Light blue dots show licks to artificial saliva. (**A**) shows taste response that occurred over all five licks of a taste trial. (**B**) shows taste responses that occurred lick-by-lick. Note the comparisons with artificial saliva (**A**,**B)** and with dry licks (**B**).

**Figure 3:**
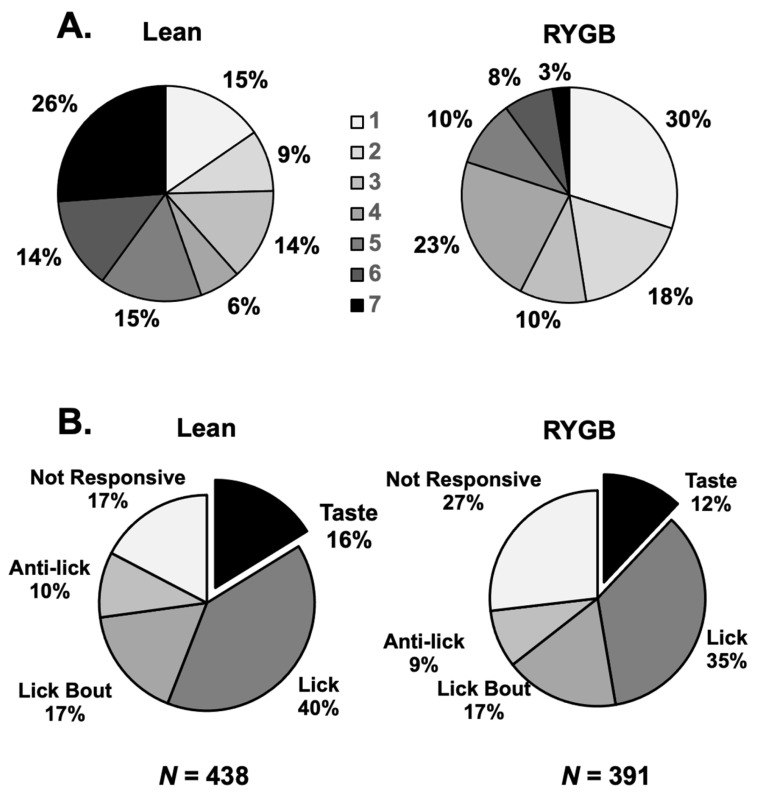
(**A**) Breadth of tuning of NTS taste-responsive cells in Lean (left) and RYGB (right) rats. Shown are the proportion of cells that respond to a single taste stimulus of the array versus two stimuli in the array, three stimuli, etc., up to the proportion of cells that responded to all seven taste stimuli. These distributions in Lean and RYG rats were statistically different (chi-squared = 19.02, 6 df; *p* = 0.004). (**B**) Proportion of cells in the various categories of NTS cells in Lean (left) and RYGB (right) rats. The incidence of taste-responsive cells in the NTS of each group of rats was comparable and the overall distributions were not statistically different (chi-squared = 3.230, df = 4, *p* = 0.520).

Taste responses occurred over two time scales. First, responses could be measured over the full five-lick stimulus trial interval (Figure 2A). We refer to this type of response as a “5-Lick” (5L) response. Second, some taste responses were not apparent over five licks but were instead characterized by brief bursts of firing following every individual taste stimulus lick (Figure 2B). These were “Lick-by-Lick” (LXL) responses. Of the 69 taste-responsive cells in lean rats, 29 (42%) showed only LXL responses. In taste cells from RYGB rats, a similar percentage of cells (21 of 47, 45%) showed only LXL taste responses. However, when responses to tastants that were tested in both lean and RYGB groups were compared, i.e., seven taste stimuli, there were 149 responses out of 483 potential 5L responses (31%) in lean rats but only 51 responses out of 329 potential 5L responses (16%) in RYGB rats. Similarly, there were proportionately fewer LXL responses (28%) in RYGB rats compared with responses in lean rats (45%). Table 1 also shows that each of the stimuli tested evoked proportionately fewer responses in taste cells in RYGB compared with lean rats. Not surprisingly, taste cells in RYGB rats responded to fewer taste stimuli that were tested than those in lean rats, as shown in Figure 3A (Chi square = 19.02, df = 6; *p* = 0.004). Collectively, these data suggest that taste stimuli were less effective in driving taste cells in RYGB rats compared with lean rats. 

**Figure 4:**
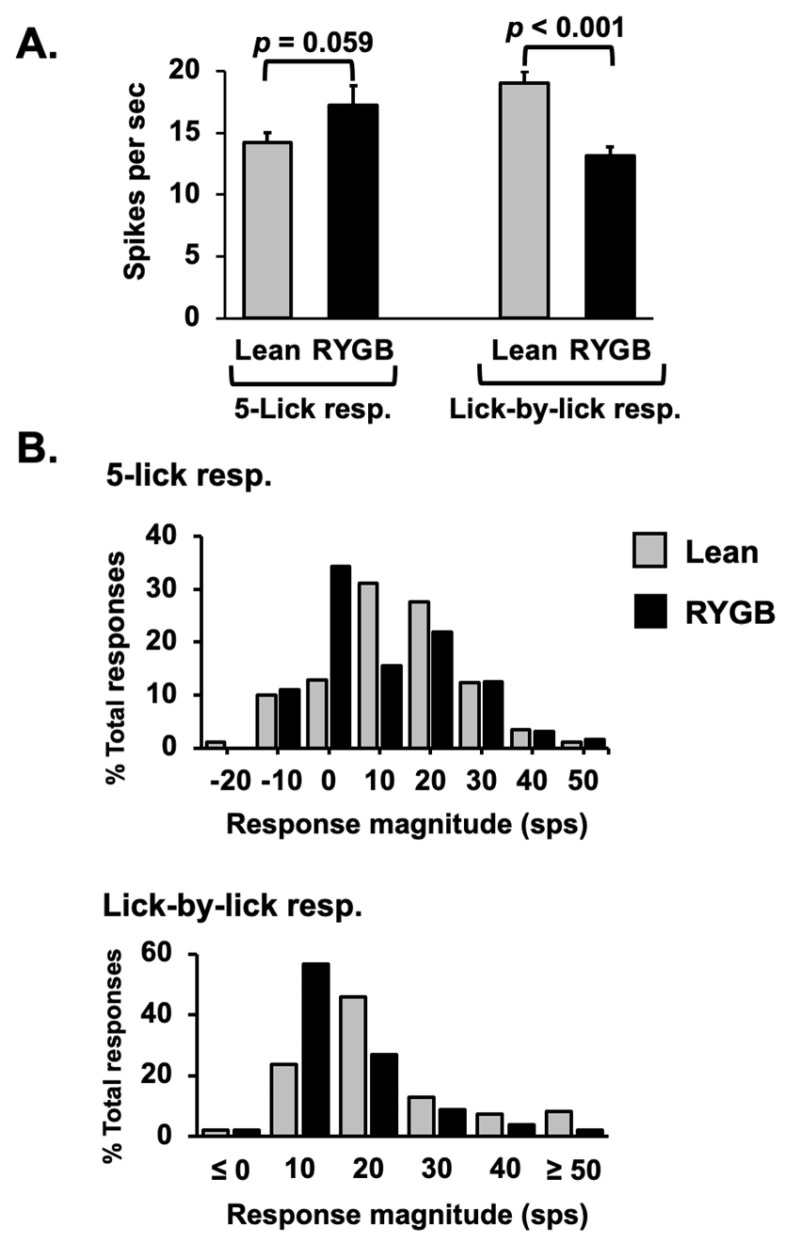
(**A**) Average excitatory NTS taste response magnitudes for Lean and RYGB cells across all stimuli. 5L lean, *n*= 129, RYGB *n* = 50 responses; LXL Lean, *n* = 241, RYGB *n* = 151. (**B**) Distribution of response magnitudes across the population of taste-responsive cells.

**Figure 5:**
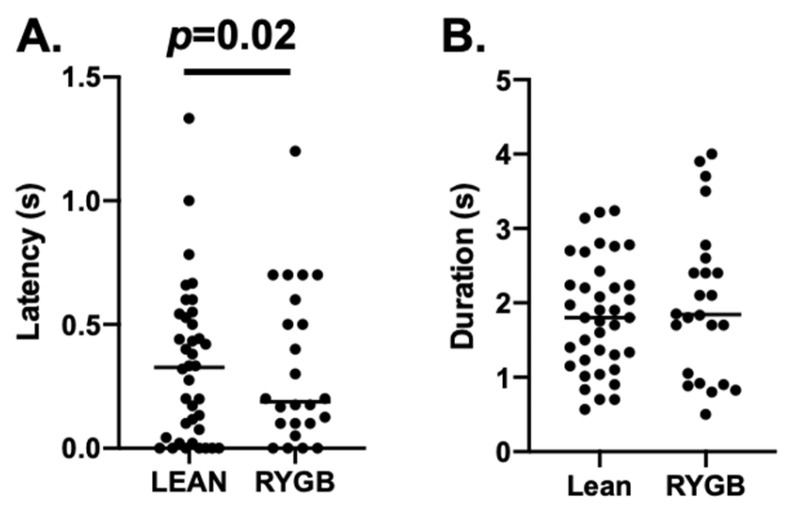
(**A**) Median latency for Lean = 0.327; RYGB = 0.188; Mann–Whitney U test *p* = 0.020 (**B**) Median duration for Lean = 1.800 s; RYGB = 1.842 s. Only 5L responses were included.

Comparisons of taste-response measures showed that 5L responses were generally similar in RYGB vs. Lean rats for any tastant, but LXL responses were weaker. For example, there were no differences in 5L response magnitudes evoked by individual tastants in RYGB vs. Lean rats (two-way ANOVA, *F* (1, 122) = 0.767, *p* = 0.383), as shown in Table 2. However, LXL responses were smaller in RYGB rats compared with those in Lean rats (two-way ANOVA, *F* (1, 280) = 19.17, *p* < 0.001). When all 5L responses were averaged, no differences in response magnitude between RYGB and Lean rats were apparent (see Figure 4A left, *t* (177) = 1.9, *p* = 0.059); however, LXL responses were significantly smaller in RYGB vs. Lean rats (Figure 4A right *t* (390) = 4.69, *p* < 0.001). The distributions of 5L response magnitudes are comparable for RYGB and Lean rats, but the distribution of LXL response magnitudes in RYGB rats shows a leftward shift compared to that of Lean rats, reflecting the weaker responses (Figure 4B). The latencies of 5L responses were significantly shorter (by about 100 ms; median latency for 5L responses in Lean rats = 0.327; for RYGB rats = 0.188 ms; Mann–Whitney U test *p* = 0.02) in RYGB than in Lean rats, but there was a good deal of overlap (Figure 5).

Because of the wide distribution of the percent body fat in RYGB rats, we divided them into those rats with percent body fat comparable to Lean rats (11–16%) and those with higher percent body fat compared with Lean rats (>16%) to examine whether the effects of RYGB surgery on taste-response magnitude varied according to percent body fat. There were 27 cells from four Lean animals and 10 cells from five animals in the RYGB group that had between 11–16% body fat. Comparison of the LXL responses (there were too few 5L responses to enable meaningful analyses) from this subset of rats showed that responses from RYGB rats were significantly larger than those in comparable Lean rats (Lean rats mean = 15.1 ± 1.1 sps, *n* = 65; RYGB rats mean = 20.0 ± 2.3 sps, *n* = 25; two-way ANOVA, *F* (1,80) = 5.229; *p* = 0.025). There was no significant interaction between the surgical group (Lean vs. RYGB) and taste stimulus (*F* (2, 80) = 0.499; *p* = 0.776), so none of the differences in response magnitude could be attributed to any one or subset of tastants. For the remining RYGB rats, that is, those with percent body fat >16%, LXL responses were significantly smaller than those in Lean rats (Lean rats mean = 19.23 ± 0.95, *n* = 213; RYGB rats mean = 9.44 ± 0.67, *n* = 66; two-way ANOVA, *F* (1, 265) = 29.93, *p* < 0.0001) with no significant group by tastant interaction (*F* (6, 265) = 0.187; *p* = 0.980). In all, RYGB rats with body composition similar to that of Lean rats showed more normalized taste responses than those RYGB rats with higher percent body fat. This suggests that RYGB rats with higher percent body fat were largely responsible for the effects of RYGB surgery on NTS taste responses.

Electrophysiological recordings from the NTS in RYGB rats showed the same heterogeneity of firing patterns as have been recorded [25,27]. That is, in addition to taste-responsive cells, there were lick cells, lick-bout cells, anti-lick cells, and non-responsive cells. The firing rate in lick cells rhythmically followed the lick pattern with peak firing bursts at different times in the lick cycle depending on the cell [37]. Anti-lick cells were active until the lick bout began and then became relatively quiescent while the rat was licking, whereas lick bout cells showed the opposite pattern, i.e., they increased their firing rate when the rat began to lick but decreased their firing rate when the rat stopped licking. The relative frequencies of these styles of activity were not statistically different between RYGB and Lean rats, as shown in Figure 3B (chi-squared = 3.230, df = 4, *p* = 0.5201; RYGB *N* = 391 cells; Lean *n* = 438 cells). Figures showing examples of these cell types can be found in [25].

### 3.3. Lick-Related Firing and Lick Behavior

**Figure 6:**
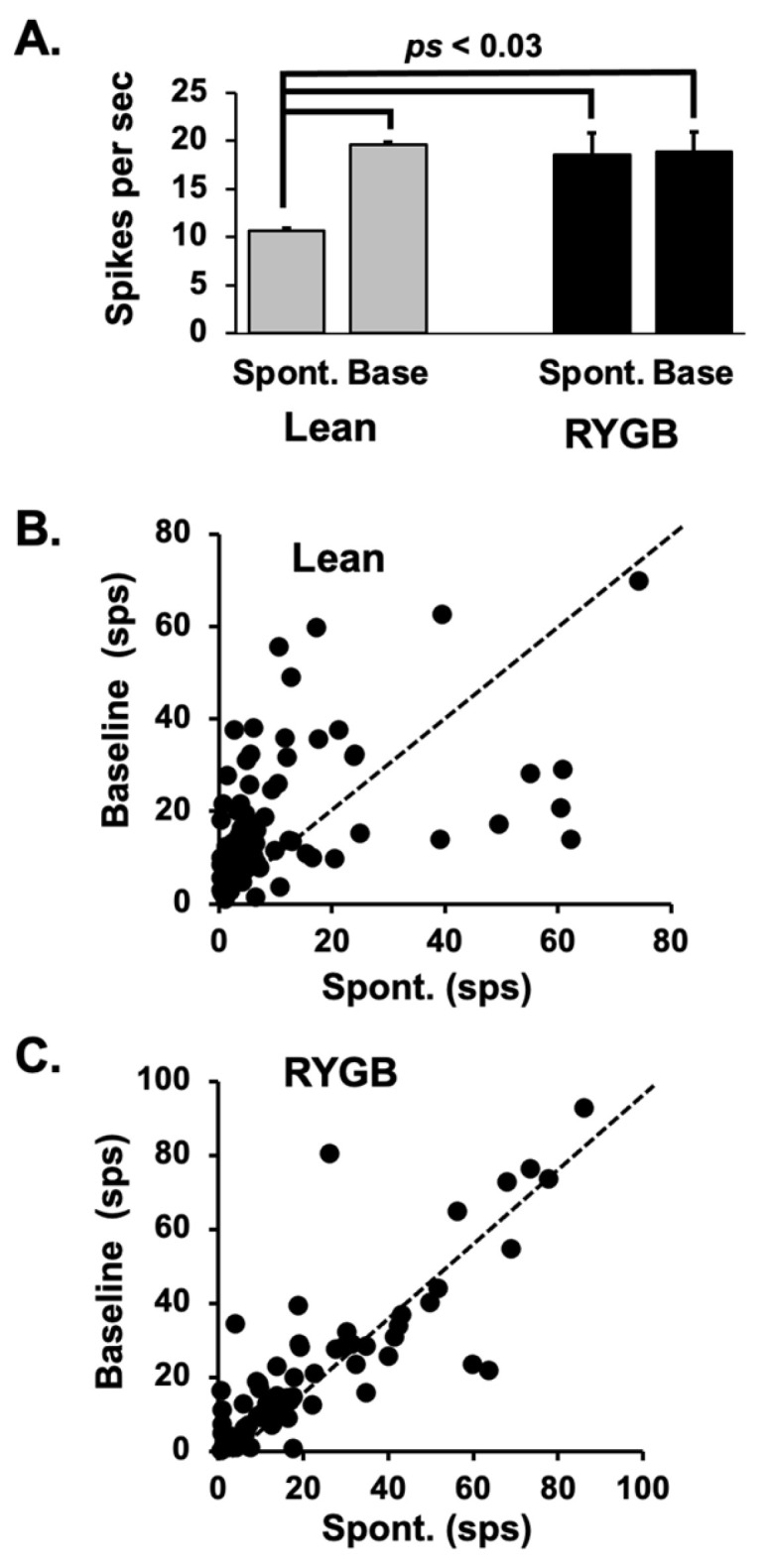
(**A**) Mean ± SEM firing rates during spontaneous (no licking) and baseline (the 1 s just prior to a taste trial) across cells in Lean and RYGB rats. Baseline firing rates in Lean rats and both spontaneous and baseline firing rates in RYGB rats were significantly greater that spontaneous firing rates in Lean rats. See text for details. (**B**,**C**) Scatterplots of individual average spontaneous (*x*-axis) vs. average baseline (*y*-axis) firing rates in Lean and RYGB rats. Dotted line indicates when spontaneous and baseline firing rates are equal. In Lean rats, baseline firing rates exceeded spontaneous firing rates in most cases, whereas baseline and spontaneous firing rates were roughly equivalent in the NTS of RYGB rats.

In most taste-responsive cells in Lean rats, the firing rate increases above spontaneous levels as the animal begins a lick bout; however, this was not the case with taste cells in RYGB rats. Figure 6 illustrates this point. Average spontaneous firing rate was calculated based on multiple 14 s intervals during which the rat was not licking. The first three seconds of this interval were excluded from the analyses since they may have included taste-evoked activity; the last one second of activity was also excluded since it might have contained activity that reflected preparation to lick. Spontaneous firing rates were significantly greater in taste cells from RYGB rats compared with those in Lean rats (*t* (104) = 4.042, *p* < 0.0001). Average baseline firing rates were based on the firing rate in the 1 s just prior to the first taste stimulus lick, averaged across all taste stimulus trials. Mean baseline firing rates did not differ between taste cells in RYGB rats vs those in Lean rats (*t* (104) = 0.441, *p* = 0.66). Thus, taste cells in RYGB rats were more spontaneously active than taste cells in Lean rats and did not react to the onset of licking by further increasing their firing rate as did cells in Lean rats. 

Table 3 shows the results of analyses of licking behavior in Lean and RYGB rats. In general, RYGB rats licked taste stimuli at a slower pace than Lean rats, as illustrated by a significantly longer (by ~20–30 ms) inter-lick interval (Wilcoxon matched-pairs signed rank test, *p* = 0.008). In addition, RYGB rats paused more often during the taste stimulus presentation but did not pause for longer times than Lean rats (Wilcoxon matched-pairs signed rank test, *p* = 0.195). Further, RYGB rats took longer to complete the five-lick taste stimulus trial (Wilcoxon matched-pairs signed rank test, *p* = 0.008), likely due to the greater frequency of within-trial pauses. Not surprisingly, RYGB rats acquired fewer taste stimulus trials within a session.

**Figure 7:**
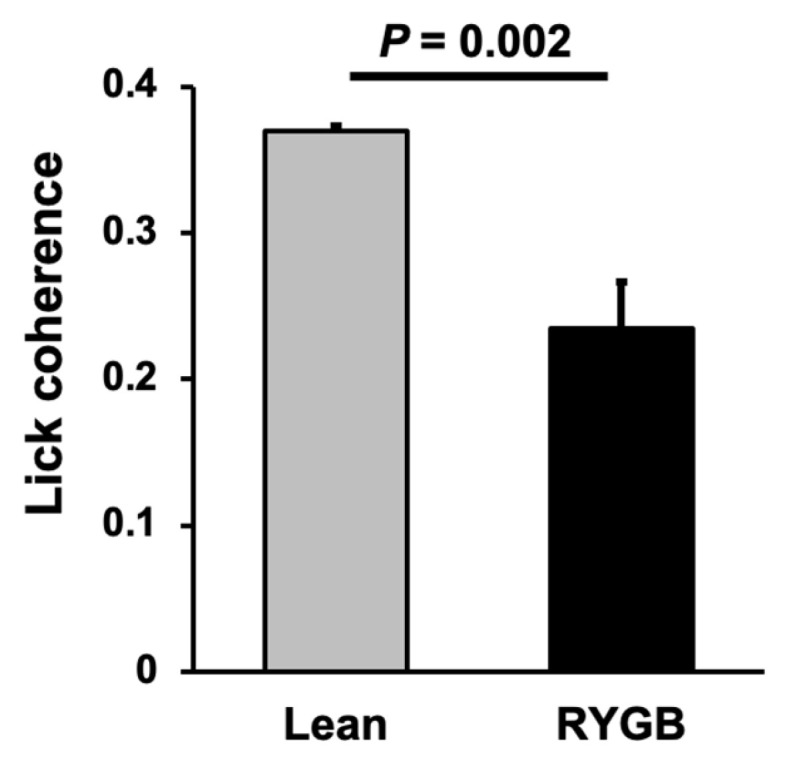
Lick coherence among taste cells. Lean *N* = 67; RYGB *N* = 41 taste cells; t = 3.120, df = 106, *p* = 0.0023, unpaired *t* test.

Taste-responsive cells in the NTS of RYGB rats were less-tightly coupled to the lick pattern than were taste-responsive cells in the NTS of Lean rats. This was evidenced by significantly less lick coherence in NTS taste cells in RYGB rats compared with those from Lean rats (Figure 7; Lean *n* = 67; RYGB *n* = 41 taste cells; *t* (106) = 3.12, *p* = 0.002). 

## 4. Discussion

Electrophysiological recordings from 47 taste-responsive cells in the NTS of awake, freely licking rats fed an HED and who had undergone RYGB surgery were compared with 69 taste-responsive cells recorded from Lean, unoperated rats. An additional 344 cells in RYGB rats (total *N* = 391) and 367 cells in Lean rats (total *N* = 436) that were not taste-responsive were also recorded. Although there was a comparable proportion of taste-responsive cells in RYGB and Lean rats, taste cells in RYGB rats were more narrowly tuned across tastants. Accordingly, there were proportionately fewer responses to individual taste stimuli across the population than in Lean rats. In addition, 5L taste responses were similar in magnitude to those in Lean rats, but LXL responses were significantly smaller. NTS taste responses in RYGB rats occurred at a shorter latency than those in Lean rats but were similar in duration. Spontaneous firing rates in NTS taste-responsive cells in RYGB rats were significantly larger than in Lean rats but equivalent to baseline firing rates. Since baseline firing rates occur while the animal is licking (but not licking tastants), the increase in average firing rate from spontaneous to baseline in Lean rats indicates a reaction to licking not seen in RYGB rats. Taste-responsive cells in RYGB rats also showed significantly less coherence with the lick pattern compared with taste cells in Lean rats. This apparent disconnect between NTS activity and the lick pattern in RYGB rats was perhaps reflected in a sluggish lick rate. These rats paused more frequently during tastant trials and took longer to complete those trials than Lean rats. In all, these data show that consumption of an HED after RYGB surgery can significantly impede, but not entirely block, RYGB-induced recovery of taste coding in the brainstem. 

### 4.1. RYGB Surgery Only Partially Ameliorated the Effects of Diet-Induced Obesity (DIO) on NTS Taste Responses

To isolate the effects of the HED from the effects of RYGB surgery, rats were maintained on an HED both before and after RYGB surgery for the duration of the experiment. Not surprisingly, then, rats in the RYGB group weighed more and had a greater percent body fat than rats in the Lean group. However, the percentage of body fat in RYGB rats fed a diet of 60 kcal% fat (18.5 ± 1.6) was significantly lower than that of DIO rats fed a diet of 45 kcal% fat (25.3 ± 2.9; *t* = 2.223, df = 2, *p* = 0.037; [24]), despite no significant difference in body weight (mean body weight for RYGB rats = 657.5 ± 25.9 gms; for DIO rats = 649.0 ± 38.9 gms). This result suggests that RYGB surgery had a protective effect on the diet-induced accumulation of body fat despite the burden of a diet high in fat following surgery.

Measures of taste coding in the NTS of rats following RYGB surgery were generally more like those in DIO rats than those in Lean rats. For example, as in DIO rats [25], LXL taste responses in RYGB rats were significantly smaller than those in Lean rats, but 5L response magnitudes were not different. Taste-responsive cells in both RYGB and DIO rats were more narrowly tuned than those in Lean rats [25]. However, in DIO rats, taste responses occurred at longer latencies and were shorter in duration than in Lean rats [25], but this was not true of taste response in RYGB rats. In addition, the proportion of taste-responsive neurons in the population was larger in DIO rats than in Lean rats, but this proportion was comparable in RYGB and Lean rats. The inflated proportion of taste-responsive cells in DIO rats was suggested to be a compensatory mechanism to offset the smaller response magnitudes seen in DIO vs Lean rats [25]. In contrast, the normalization of the proportion of tase-responsive cells along with smaller responses magnitudes and narrower tuning in RYGB vs. Lean rats suggests that taste stimuli evoke a weaker population signal in the NTS in RYGB rats. That is, when a given taste stimulus is on the tongue, fewer NTS cells respond with weaker increases in firing rate in RYGB rats vs. Lean rats.

In both DIO and RYGB rats, attenuated NTS taste responses may reflect a compromised input from the tongue [38]. Kauffman et al. [38] found that there were fewer taste buds on the tongue of mice fed an HED. The same deficit was found in rats fed an HED, and it persisted even after rats were switched to a standard chow diet for several months [39]. In contrast, Hyde et al. [40] found that DIO female rats maintained on an HED following RYGB surgery did not show any deficits in the number of taste pores in the circumvallate papillae of the tongue. When there are fewer taste buds, a given tastant would evoke a weaker response in taste-related peripheral nerves in DIO and RYGB than in Lean rats. This weakened input would be naturally reflected in a weaker response in the NTS. The observation that RYGB rats show proportionately fewer and smaller taste responses in the NTS than either DIO or Lean rats likely reflects a combination of the effects of a HED and the surgery. 

Changes in licking-related neural activity and in licking behavior were generally similar in RYGB and DIO rats, but different from that in Lean rats. For example, when Lean rats initiate a lick bout, the average firing rate of taste-responsive NTS cells increases, but this was not the case for cells in both RYGB and DIO [25] rats. That is, in the NTS of RYGB and DIO rats, spontaneous and baseline firing rates were not different, though the spontaneous firing rate of NTS taste cells in RYGB rats was higher than that in Lean rats, unlike the spontaneous firing rate in DIO rats [25]. 

The observed increase in spontaneous firing rate of the NTS taste-responsive cells of the RYGB rats is consistent with an increase glutamatergic drive. Previous studies demonstrated that transient withdrawal of vagal afferents, due to an unavoidable damage of the gastric vagal branches during the RYGB surgery [7,41,42], may result in neuronal plasticity withing the NTS [7,41,42]. In support of this hypothesis, neurons in the dorsal vagal complex (the efferent vagal brain region), which is one glutamatergic synapse away from the NTS, also showed increased excitability [43], an effect that was implicated in recovered vago-vagal regulation following RYGB [44]. An alternative hypothesis is that partial vagal denervation of the stomach, paradoxically, causes RYGB subjects continue to experience satiation [45]. The behavioral relevance of increased spontaneous firing rate of NTS taste-responsive neurons, when compared to an unaltered or even reduced response magnitude, is a reduction in information transfer to the next neuron population within the taste and reward circuitries.

In addition to the muted responsiveness to lick bouts, there was a significantly lower average lick coherence in NTS cells in both RYGB and DIO [25] rats compared with that in Lean rats. Moreover, RYGB rats showed a slower lick rate, evidenced by ~20 ms increase in the interlick interval, compared with that in Lean rats. RYGB rat also took more pauses during the taste stimulus trials and took longer to complete the five-lick stimulus trails than lean rats. These results were similar to those in DIO rats [25]. When they occurred, pauses in licking in both RYGB and DIO [24] rats were not any longer than those in Lean rats. Collectively, these data suggest that differences between RYGB and Lean rats with respect to lick-related NTS activity and licking behavior may be due to the effects of a chronic HED. However, without a comparison to DIO rats that had undergone RYGB surgery and were subsequently maintained on a standard chow diet, this conclusion remains speculative.

### 4.2. Relation of the Effects of RYGB Surgery on Changes in Taste Preference

The hallmark of behavioral outcomes following RYGB surgery is improved food preferences characterized by healthier food choices due to reduced intake of highly sweet and fatty meals [46,47]. Blunted preferential intake of sweet and fatty fluids have been demonstrated in bypass patients as well as in rat models of RYGB [48,49,50]. In fact, improved taste/food preferences are predictors of durable weight loss following this procedure [51,52]. In addition, RYGB also results in resolution of food addictions [53].

One mechanism that might explain altered food preferences and food cravings following RYGB surgery is changes in taste function, as documented here in the brainstem. Some human studies [16,54,55,56,57], as well as our animal research, have confirmed such effects [34], whereas other findings are more equivocal [40,58]. However, it is possible that changes in food reward, rather than taste sensations, may explain the beneficial outcomes of RYGB surgery [59]. Recent human studies have provided support for this notion [60,61,62,63] and also showed that RYGB reduces hedonic responses to high-calorie foods in brain reward areas [64]. Alternatively, recent animal studies have shown increased conditioned food aversions [65] to repeated exposure to strong sweet and fatty foods. However, most of these models of RYGB used lean rats, thereby negating the influence of previous exposure to obesogenic diets, or the complexity from factors related to obesity. Nevertheless, we may assume those aspects of taste coding that are recovered after RYGB in our study may contribute to those altered food preferences. 

### 4.3. Limitations of the Present Study

The present work must be considered in the context of several possibly impactful limitations. For example, we did not measure hormonal changes or microbiome changes in the RYGB rats to confirm the efficacy of the procedure. Considering the large variation in the percent body fat among RYGB rats, percent body fat may have enabled predictions of changes in taste responses. As we have described, the bulk of the alterations in taste responsivity in RYGB vs. Lean rats derived from RYGB rats with high percent body fat owing no doubt to the HED. Also, while our main comparison group with RYGB rats were lean rats, a group of sham-operated or DIO rats maintained on the same HED as the RYGB rats might have served as better controls. Finally, another important caveat to the present results is that the RYGB subjects were all male, whereas the great majority of RYGB surgery in humans is done in females [66]. In addition, there are known sex differences in taste detection and preference [67]. Clearly, these limitations call for additional studies to determine the underlying variables that can fully account for the present results.

## 5. Conclusions

The present study showed that RYGB surgery normalized the proportion of taste-responsive NTS cells that was inflated in DIO rats. Those taste responses that were present did not show either the breadth of tuning across tastants nor the vigor of responses recorded in Lean rats. While these effects are likely due to the effects of a chronic HED, the findings shed light on the effect of surgery on primary taste coding in the hindbrain that may explain improvements in taste-guided behaviors, e.g., taste preferences and aversions that helps patients to reduce intake of highly stimulating ‘junk’ foods.

An important caveat to the present results is that the subjects were all male, whereas the great majority of RYGB surgery in humans is done in females [66]. In addition, there are known sex differences in taste detection and preference [67]. 

## Figures and Tables

**Table 1:** Percent taste responses (number) in Lean and RYGB rats.

	5-Lick		Lick-By-Lick	
Stimulus	Lean (*n* = 40)	RYGB (*n* = 26)	Lean (*n* = 61)	RYGB (*n* = 38)
0.5 M Sucrose	40% (16)	31% (8)	46% (28)	24% (9)
0.1 M Sucrose	70% (28)	23% (6)	53% (32)	34% (13)
0.05 M Sucrose		27% (7)		34% (13)
100% Grape Juice	70% (28)	46% (12)	62% (38)	50% (19)
25% Grape Juice	63% (25)	35% (9)	57% (35)	45% (17)
12.5% Grape Juice		31% (8)		26% (10)
0.1 M NaCl		35% (9)		40% (15)
0.05 M NaCl	38% (15)	35% (9)	48% (29)	26% 910)
75% Clam Juice	55% (22)	35% (9)	53% (32)	37% (14)
0.1 M MSG + 0.01 M IMP		23% (6)		32% (12)
25% Cream	33% (13)	19% (5)	39% (24)	29% (11)
Artificial Saliva	28% (11)	23% (6)	46% (28)	32% (12)

**Table 2:** NTS taste response magnitude in Lean and RYGB rats. Mean ± SEM (*N*).

A. 5-Lick				
	Excitatory		Inhibitory	
Stimulus	Lean	RYGB	Lean	RYGB
0.5 M Sucrose	15.1 ± 2.6 (12)	24.9 ± 3.7 (4)	−12.2 ± 1.3 (4)	−4.9 ± 0.9 (3)
0.1 M Sucrose	11.6 ± 1.9 (23)	21.4 ± 3.4 (4)	−10.0 ± 1.9 (5)	−8.0 (2)
0.05 M Sucrose		25.4 ± 4.5 (4)		−6.1 ± 1.8 (3)
100% Grape Juice	15.5 ± 2.2 (21)	15.5 ± 3.4 (6)	−15.2 ± 2.9 (7)	−9.8 ± 1.8 (5)
25% Grape Juice	14.2 ± 2.5 (20)	11.7 ± 3.0 (5)	−11.4 ± 2.6 (5)	−5.7 ± 1.5 (4)
12.5% Grape Juice		20.7 ± 7.6 (4)		−7.5 ± 2.5 (4)
0.1 M NaCl		20.4 ±8.1 (5)		−8.0 ± 1.9 (7)
0.05 M NaCl	15.2 ± 2.8 (12)	11.5 (2)	−11.1 ± 2.7 (3)	−6.6 ± 1.3 (3)
75% Clam Juice	13.6 ±1.6 (4)	18.2 ± 7.0 (5)	−12.7 ± 2.6 (4)	−6.0 ± 1.6 (3)
0.1 M MSG + 0.01 M IMP		5.4 (2)		−7.1 ± 2.0 (5)
25% Cream	18.7 ± 2.3 (13)	13.7 ± 4.1 (5)	−8.2 (2)	(0)
Artificial Saliva	12.2 ± 1.7 (10)	11.6 ± 2.3 (4)	−13.4 (1)	−6.3 (1)
**B. Lick-By-Lick**				
	**Excitatory**		**Inhibitory**	
**Stimulus**	**Lean**	**RYGB**	**Lean**	**RYGB**
0.5 M Sucrose	18.8 ±2.7 (27)	15.2 ± 3.3 (9)	−9.8 (1)	(0)
0.1 M Sucrose	16.9 ± 2.2 (31)	12.8 ± 2.4 (12)	−29.6 (1)	−12.6 (1)
0.05 M Sucrose		13.2 ± 2.8 (11)		−7.6 (2)
100% Grape Juice	21.2 ± 2.3 (36)	12.3 ± 2.3 (19)	−30.1 (2)	(0)
25% Grape Juice	19.3 ± 2.5 (35)	11.8 ± 1.8 (17)	(0)	(0)
12.5% Grape Juice		14.0 ± 2.9 (9)		−9.8 (1)
0.1 M NaCl		13.6 ± 3.1 (15)		(0)
0.05 M NaCl	20.5 ± 2.8 (28)	9.6 ± 1.6 (10)	−31.5 (1)	(0)
75% Clam Juice	20.4 ± 2.8 (32)	17.1 ± 2.9 (14)	(0)	(0)
0.1 M MSG + 0.01 M IMP		16.5 ± 3.0 (12)		(0)
25% Cream	16.4 ± 2.1 (24)	10.2 ± 1.4 (11)	(0)	(0)
Artificial Saliva	17.8 ± 2.4 (28)	11.6 ± 1.5 (12)	(0)	(0)

**Table 3:** Lick behavior in Lean and RYGB rats.

	ILI (s)			No. Pauses		Pause Length (s)	
Stimulus	Lean		RYGB	Lean	RYGB	Lean	RYGB
0.5 M Sucrose	0.153		0.170	2	19	1.165	1.342
0.1M Sucrose	0.151		0.170	2	22	1.090	1.430
0.05 M Sucrose			0.170		24		1.560
100% Grape Juice	0144		0.161	27	119	1.586	1.570
25% Grape Juice	0.150		0.170	5	84	1.230	1.360
12.5% Grape Juice			0.170		57		1.637
0.1 M NaCl			0.169		23		1.330
0.05 M NaCl	0.152		0.179	0	57	N/A	1.680
75% Clam Juice	0.150		0.170	1	31	1.900	1.282
0.1 M MSG + 0.01 M IMP			0.160		18		1.290
25% Cream	0.160		0.180	3	58	1.448	1.906
Artificial Saliva	0.160		0.180	4	35	1.728	1.950
	**Time to Complete 5L (s)**			**No. Trials**			
**Stimulus**	**Lean**	**RYGB**	**Lean**	**RYGB**			
0.5 M Sucrose	0.627		0.690	1408	643		
0.1 M Sucrose							
0.05 M Sucrose			0.170		24		
100% Grape Juice	0144		0.161	27	119		
25% Grape Juice	0.150		0.170	5	84		
12.5% Grape Juice			0.170		57		
0.1 M NaCl			0.169		23		
0.05 M NaCl	0.152		0.179	0	57		
75% Clam Juice	0.150		0.170	1	31		
0.1 M MSG + 0.01 M IMP			0.160		18		
25% Cream	0.160		0.180	3	58		
Artificial Saliva	0.160		0.180	4	35		

## Data Availability

Thedata contained in the istudy are publicly available at FigShare at https://doi.org/10.6084/m9.figshare.21266181.v1.
